# Brazilian science through the looking glass: a scientometric perspective from within and beyond

**DOI:** 10.1590/1414-431X2026e15002

**Published:** 2026-04-27

**Authors:** A. Sá-Nunes, Y.M. Pessoa-Gonçalves, C.J.F. Oliveira

**Affiliations:** 1 Laboratório de Imunologia Experimental, Departamento de Imunologia, Instituto de Ciências Biomédicas, Universidade de São Paulo, São Paulo, SP, Brasil; 2 Laboratório de Imunologia e Ciências Ômicas - LImCOm, Departamento de Microbiologia, Imunologia e Parasitologia, Instituto de Ciências Biológicas e Naturais, Universidade Federal do Triângulo Mineiro, Uberaba, MG, Brasil

**Keywords:** Brazilian science, Scientometrics, Citation analysis, Research support, Science policies

## Abstract

Citation analysis has emerged as a key area in scientometrics. However, the global movement toward open science, alongside the pervasive “publish or perish” culture, underscores the need to reevaluate the paradigm of citations as a measure of impact and quality. Brazil, a top 15 producer of scientific articles, has established the Lattes Platform, a comprehensive resource data on virtually active researchers in the country. Herein, the Brazilian scientific landscape was analyzed by integrating a widely used global ranking based on large-scale citation metrics with individual-level data from the Lattes Platform. The analysis assessed the impact, distribution, and disparities of Brazilian science across disciplines, geographic regions, and institutional affiliations from 2019-2023. Results showed that Brazilian researchers account for approximately 0.43% of the world's most cited scientists, a significant underrepresentation relative to Brazil's population share and scientific potential. Most highly cited scientists are concentrated in three states within the Southeast region, reflecting longstanding economic and infrastructural advantages. The majority of top Brazilian scientists work in Life Sciences, with particular representation in the subfields Zoology, Tropical Medicine, and Mycology & Parasitology. While Brazil's scientific output compares favorably with other South American and African countries, it remains behind nations with higher gross domestic products per capita and Human Development Index. Nonetheless, 73% of the most cited researchers receive national Research Productivity Grants, indicating a positive correlation between citation and qualified scientific excellence. These findings offer a deeper understanding of Brazilian scientific production from a citation perspective and advocate for strategic policy shifts.

## Introduction

A few years ago, a large-scale database of the most cited scientists worldwide was produced and has been subsequently updated annually (1-7). Using data from Scopus, the authors devised a composite score that integrated six log-transformed indicators: I) total citations; II) Hirsch *h*-index; III) coauthorship-adjusted Schreiber hm-index; IV) citations to papers as single author; V) citations to papers as single or first author; and VI) citations to papers as single, first, or last author (8). Following the release of each year's new list, thousands of news articles and reports are produced globally, linking an individual's, department's, or institution's presence in the database to the perceived quality of their research. However, as is common with any scientometric evaluation, this ranking has faced criticism. Many productive scientists excluded from the list may disagree with the criteria used for such classifications (9-12). Additionally, there are inherent limitations and collateral effects associated with relying on ‘raw’ numbers and indices, which can be manipulated through questionable practices, including salami science, paper mills, extreme self-citation, and citation farms (13-15).

Despite the discontent that occasionally arises, we assert that, albeit imperfect, a rigorous methodology employing multiple parameters rather than a single indicator offers a reasonable inference of quality in scientific research. This approach provides a multifaceted view of the scientific impact of departments and institutions, which can guide the establishment of local and comprehensive public policies in science, technology, and innovation. The algorithms used to compile these large-scale databases and the precise sources for data retrieval are critical to the quality of the information generated. In addition, despite recent advancements in artificial intelligence, manual verification (when possible) and the human capability to discern subtle nuances remain indispensable for rectifying any eventual discrepancies present in these extensive databases. Fine-tuning such data is feasible only when detailed and standardized sources are readily available.

Even with refinements to the ranking data, it remains unclear whether these citation metrics directly or indirectly reflect the actual quality or impact of the research and science produced. In other words, the literature lacks comprehensive studies that evaluate the complexity of these data: which areas of knowledge have the largest number of researchers, how these figures compare across countries with different gross domestic products (GDP) per capita or Human Development Index (HDI), and what disparities exist within a single country based on citation patterns. Brazil, a country with vast territorial dimensions, has developed a scientific infrastructure over the past half century, but it is not highly competitive relative to its considerable global potential. Although Brazil ranks among the top 15 countries in scientific article production, the representativeness of its researchers on citation parameters remains uncertain, both domestically and in comparison with other nations.

In Brazil, a national repository called “Lattes Platform” was launched in 1999 by the National Council for Scientific and Technological Development (CNPq), an organization of the Federal Government under the Ministry of Science, Technology, and Innovation. The Lattes Platform has emerged as the primary scientific information system in the country. It hosts the *Curriculum vitae* (Lattes CV) of virtually all active researchers in Brazil, from undergraduate students to well-established scientists (https://lattes.cnpq.br) (16). The Lattes Platform not only provides individual information on researchers' affiliations, grant approvals, supervision records, and bibliographical, technical, and artistic production but also serves as a historical and integrated directory of all officially recognized research groups in the country. In broader terms, the Lattes Platform has become an indispensable resource for evaluating grant applications, job candidacies, career advancements, and the selection of students for graduate programs, among numerous other applications. Additionally, the Lattes CV plays a vital role in assessing the scientific output of leading Brazilian researchers who are awarded Research Productivity Grants (PQ, for short) by the federal government (17).

By combining the large-scale database of the most cited scientists with individual information from the Lattes Platform, we successfully assembled a detailed profile of active Brazilian researchers and their affiliations. This enabled us to present a snapshot of the nation's scientific landscape and, based on several parameters, compare it with other countries at different stages of development. In addition, tracking the geographic locations of Brazilian-based researchers, along with the fields and subfields of their publications, allowed us to explore the virtues and weaknesses of various research communities within Brazil based on citation data. Our analysis highlights the stark inequalities present in the country while also revealing promising surprises - especially in light of the urgent need for robust and strategic science policies.

## Material and Methods

The databases used in this study are publicly available in the Mendeley repository in Excel format (https://doi.org/10.17632/btchxktzyw.1, https://doi.org/10.17632/btchxktzyw.2, https://doi.org/10.17632/btchxktzyw.3, https://doi.org/10.17632/btchxktzyw.5, https://doi.org/10.17632/btchxktzyw.6, https://doi.org/10.17632/btchxktzyw.7), derived from Scopus data provided by Elsevier through ICSR Lab (https://www.elsevier.com/icsr/icsrlab). The spreadsheets compile the 100,000 most-cited authors (including and excluding self-citations) across 20 scientific fields and 174 subfields, and also include the top 2% of authors in each subfield. The ranking is based on a composite indicator that incorporates six citation metrics: total citations; Hirsch *h*-index; coauthorship-adjusted Schreiber hm-index; number of citations to papers as single author; number of citations to papers as single or first author; and number of citations to papers as single, first, or last author (8).

These spreadsheets represent the citation impact for a single calendar year: 2019 (SCY19), 2020 (SCY20), 2021 (SCY21), 2022 (SCY22), and 2023 (SCY23), along with the career-long citation impact spanning from 1960 to 2019 (CL19), 1960 to 2020 (CL20), 1960 to 2021 (CL21), 1960 to 2022 (CL22), and 1960 to 2023 (CL23). Data from Brazilian-based scientists were extracted by filtering the option ‘bra’ (the international alpha-3 code for Brazil according to ISO 3166 standard) in column ‘C’ of each spreadsheet. This process yielded 853 records from 161,441 scientists for SCY19; 1,132 records from 190,063 scientists for SCY20; 1,212 records from 200,409 scientists for SCY21; 1,294 records from 210,198 scientists for SCY22; and 1,340 records from 222,454 scientists for SCY23. Similarly, for the career-long impact, there were 600 records from 159,683 scientists for CL19; 812 records from 186,177 scientists for CL20; 844 records from 195,605 scientists for CL21; 979 records from 204,643 scientists for CL22; and 1,077 records from 215,908 scientists for CL23.

An extensive manual systematic validation of the entire database also revealed that a few Brazilian scientists were not included in the original list. In addition, a few foreign scientists were mistakenly affiliated with Brazilian institutions, likely due to former or ongoing collaborations (e.g., visiting scholars or professors, temporary and/or concomitant work contracts) that were not detected by the algorithm that generated the large-scale database. The overall error rates identified through manual inspection, compared to the original database, were 3.09% for CL19, 2.65% for CL20, 3.05% for CL21, 3.47% for CL22, 3.43% for CL23, 2.03% for SCY19, 1.16% for SCY20, 1.68% for SCY21, 1.85% for SCY22, and 2.16% for SCY23. This analysis confirmed the robustness of the algorithm used by the lists' creators, as manual checks detected less than 4% errors in the affiliations and countries of scientists included in the database.

The public Lattes CV of each Brazilian scientist was individually accessed for the respective calendar year corresponding to the published lists. For each listed scientist, the following information was recorded: institution type (public or private), geographic region, state, and municipality of the institutional affiliation. The original database columns were maintained, including: I) top-ranked higher-level Science-Metrix category (field and subfield 1); and II) the second-ranked Science-Metrix category (subfield 2). The PQ category was updated according to data achieved through the federal government transparency portal (https://www.gov.br/acessoainformacao/pt-br).

Since institutional affiliations can be expressed in different ways in English, this parameter was standardized according to the Research Organization Registry (https://ror.org/). This process enabled accurate clustering of authors based on their affiliations. In addition, since the original algorithm retrieved the institutional affiliation based on the last publication of each scientist, this information was sometimes inaccurate due to multiple affiliations stated in the authorship description. Therefore, the information underwent manual updates focused on the primary affiliation. In cases where multiple, concurrent, and equivalent affiliations were present, precedence was given to the most longstanding affiliation. When recent changes in institutional affiliation were detected, the new affiliation was considered only if the appointment was equal to or greater than two years. Whenever necessary, additional professional profiles (e.g., Publons, ORCID, Loop, Scopus, and LinkedIn) were consulted to complement any missing information about the scientists.

After manual corrections, all the data were organized in Microsoft Excel (Version 2307 Build 16.0.16626.20170) for analysis. The data are presented in Supplementary Tables S1-S10. Figures were created using QGIS (version 3.30.2), ScapeToad (version 1.1), and Adobe Photoshop CC (version 14.0).

## Results and Discussion

### The relative contribution of Brazilian-based scientists in relation to the total and by field/subfield

When analyzing the career-long (CL) citation data across the 2019-2023 period, an average of 0.43±0.04% of the records had their affiliations with Brazilian institutions confirmed after manual adjustments (Table 1). Likewise, when considering a single calendar year (SCY) from 2019 to 2023, an average of 0.577±0.03% of the records were affiliated with Brazilian institutions. Considering that Brazil accounts for approximately 2.7% of the global population (18), these figures suggest that the country underperforms in this scientometric ranking relative to its population size.

**Table 1:** Career-long and single calendar year citation impact of scientists affiliated with Brazilian institutions in the global database (2019-2023).

Year	CL (n/N)	CL (%)	SCY (n/N)	SCY (%)
2019	582/159,683	0.364	836/161,441	0.518
2020	791/186,177	0.425	1119/190,063	0.589
2021	819/195,605	0.419	1192/200,409	0.595
2022	945/204,643	0.461	1270/210,198	0.604
2023	1040/215,908	0.481	1289/222,454	0.583

Data show the number of scientists affiliated with Brazilian institutions (n) relative to the database total (N). CL: career-long citation impact; SCY: single calendar year citation impact.

Brazilian-based scientists listed in the CL and SCY databases were also evaluated according to the field and subfield of their publications. Considering the categories used in the original database, there was at least one Brazilian scientist in 17 of the 20 major fields of knowledge categorized in CL23 and SCY23. Notably, regardless of whether the assessment is based on the CL or SCY databases, the majority of Brazilian-based scientists developed their research in the Life Sciences. Over the years evaluated, six fields have consistently accounted for approximately 75% of the study areas having Brazilian representation within the CL lists (mean±SD): Clinical Medicine (26.95±1.38%), Chemistry (11.50±0.64%), Enabling & Strategic Technologies (10.71±1.30%), Physics & Astronomy (9.97±1.04%), Biology (9.72±1.04%), and Engineering (7.82±0.74%). Conversely, the percentage of Brazilian scientists varies between 0.04±0.05% and 0.38±0.09% in five other fields: Psychology & Cognitive Sciences, Philosophy & Theology, Historical Studies, Built Environment & Design, and Economics & Business. Furthermore, there are no Brazilian-based scientists represented in three fields across any of the CL lists: Communication & Textual Studies, Social Sciences, and Visual & Performing Arts (Table 2).

**Table 2:** Number of Brazil-based scientists by research field for each career-long and single calendar year.

Field	CL19	CL20	CL21	CL22	CL23	SCY19	SCY20	SCY21	SCY22	SCY23
Clinical Medicine	149 (25.60%)	217 (27.43%)	206 (25.15%)	262 (27.72%)	300 (28.85%)	236 (28.23%)	348 (31.10%)	386 (32.38%)	428 (33.70%)	441 (33.64%)
Chemistry	66 (11.34%)	89 (11.25%)	104 (12.70%)	108 (11.43%)	112 (10.77%)	79 (9.45%)	112 (10.01%)	121 (10.15%)	133 (10.47%)	135 (10.3%)
Enabling & Strategic Technologies	49 (8.42%)	88 (11.13%)	102 (12.45%)	102 (10.79%)	112 (10.77%)	64 (7.66%)	91 (8.13%)	99 (8.31%)	99 (7.80%)	111 (8.47%)
Physics & Astronomy	63 (10.82%)	79 (9.99%)	80 (9.77%)	92 (9.74%)	99 (9.52%)	74 (8.85%)	93 (8.31%)	85 (7.13%)	91 (7.17%)	84 (6.41%)
Biology	66 (11.34%)	77 (9.73%)	84 (10.26%)	84 (8.89%)	87 (8.37%)	95 (11.36%)	120 (10.72%)	131 (10.99%)	122 (9.61%)	123 (9.38%)
Engineering	52 (8.93%)	62 (7.84%)	54 (6.59%)	75 (7.94%)	81 (7.79%)	59 (7.06%)	72 (6.43%)	54 (4.53%)	73 (5.75%)	80 (6.1%)
Biomedical Research	46 (7.90%)	52 (6.57%)	51 (6.23%)	62 (6.56%)	64 (6.15%)	68 (8.13%)	74 (6.61%)	73 (6.12%)	87 (6.85%)	92 (7.02%)
Agriculture. Fisheries & Forestry	30 (5.15%)	38 (4.80%)	38 (4.64%)	52 (5.50%)	64 (6.15%)	60 (7.18%)	84 (7.51%)	99 (8.31%)	102 (8.03%)	111 (8.47%)
Information & Communication Technologies	22 (3.78%)	32 (4.05%)	48 (5.86%)	44 (4.66%)	50 (4.81%)	37 (4.43%)	40 (3.57%)	57 (4.78%)	47 (3.70%)	44 (3.36%)
Earth & Environmental Sciences	13 (2.23%)	20 (2.53%)	21 (2.56%)	27 (2.86%)	31 (2.98%)	24 (2.87%)	32 (2.86%)	28 (2.35%)	33 (2.60%)	39 (2.97%)
Public Health & Health Services	10 (1.72%)	16 (2.02%)	11 (1.34%)	14 (1.48%)	13 (1.25%)	13 (1.56%)	18 (1.61%)	16 (1.34%)	17 (1.34%)	13 (0.99%)
Mathematics & Statistics	11 (1.89%)	13 (1.64%)	15 (1.83%)	13 (1.38%)	15 (1.44%)	16 (1.91%)	18 (1.61%)	22 (1.85%)	16 (1.26%)	17 (1.3%)
Economics & Business	2 (0.34%)	4 (0.51%)	2 (0.24%)	4 (0.42%)	4 (0.38%)	6 (0.72%)	9 (0.80%)	15 (1.26%)	11 (0.87%)	11 (0.84%)
Built Environment & Design	2 (0.34%)	3 (0.38%)	1 (0.12%)	4 (0.42%)	5 (0.48%)	4 (0.48%)	4 (0.36%)	3 (0.25%)	6 (0.47%)	4 (0.31%)
Historical Studies	1 (0.17%)	1 (0.13%)	1 (0.12%)	1 (0.11%)	1 (0.10%)	1 (0.12%)	3 (0.27%)	2 (0.17%)	3 (0.24%)	3 (0.23%)
Philosophy & Theology	0	0	0	1 (0.11%)	1 (0.10%)	0	0	0	0	0
Psychology & Cognitive Sciences	0	0	1 (0.12%)	0	1 (0.10%)	0	0	0	1 (0.08%)	1 (0.08%)
Social Sciences	0	0	0	0	0	0	1 (0.09%)	0	1 (0.08%)	2 (0.15%)
Communication & Textual Studies	0	0	0	0	0	0	0	1 (0.08%)	0	0
Visual & Performing Arts	0	0	0	0	0	0	0	0	0	0
Total	582	791	819	945	1040	836	1119	1192	1270	1311

The table is sorted in descending order of CL23 (the last year evaluated) based on the number of researchers present in the field. CL: career-long citation impact; SCY: single calendar year citation impact.

Regarding the two sets of subfields in the lists (totaling 174 entries), Brazilian-based scientists had representation in 114, 122, 115, 125, and 125 subfields listed for CL19, CL20, CL21, CL22, and CL23, respectively. When comparing the number of Brazilian researchers within a particular subfield to the overall number of researchers in the same subfield across the CL lists, Brazil excelled in three subfields (mean±SD): Zoology (5.70±0.41%), Tropical Medicine (4.50±1.40%), and Mycology & Parasitology (2.57±0.71%). Additional subfields worthy of recognition included Medicinal & Biomolecular Chemistry (1.76±0.21%), Dentistry (1.67±0.23%), Analytical Chemistry (1.56±0.04%), Entomology (1.42±0.19%), Food Science (1.26±0.16%), Biotechnology (1.15±0.27%), Agronomy & Agriculture (1.09±0.11%), and Ecology (1.05±0.06%) (Supplementary Tables S11-S15).

On the other hand, while most of these subfields reflected Brazil's traditional areas of expertise, it is surprising to note our underrepresentation in other areas where Brazilian contributions are typically recognized. In at least 35 subfields, no Brazilian-based scientists have been ranked in the past five single calendar years. For instance, despite Brazil having one of the largest coastlines in the world and extraordinarily rich marine biodiversity (19), there are no Brazilian-based scientists among the highly cited researchers in the Oceanography subfield. Furthermore, Brazil was the 9th largest motor vehicle producer in 2024 (https://www.oica.net/category/production-statistics/2024-statistics/); however, no scientists from the country are listed in the subfield of Automobile Design & Engineering. This absence may stem from the fact that only foreign automobile manufacturers have production facilities in Brazil, suggesting that fundamental developmental research in this subfield is likely limited to the companies' headquarters.

Additionally, we believe that the primary reason for the lack of Brazilian representation in many subfields related to the humanities, economy, and culture is the tendency for scholarly literature in these areas to be published in Portuguese (Brazil’s native language) or in other Latin-derived languages (such as Spanish, Italian, and French) rather than in English. The reasons for this are diverse and may vary across different subfields, including linguistic and rhetorical demands, resource and network access, cultural and contextual differences, and even resistance to publishing exclusively in English. Supporting this perspective, a previous study analyzed the number of articles published in Portuguese and English in three disciplinary areas - Linguistics, Information Science and Library Science, and Pharmacology and Pharmacy - retrieved from the Web of Science over a 20-year period (1998-2017). The authors found that, although English-language papers were consistently more numerous and showed an increased trend in recent years, the growth of Portuguese-language publications has been particularly pronounced, particularly in Linguistics (20). While the scenario is gradually shifting due to the dominance of English in academic publishing, global visibility, and indexing, linguistic barriers still pose a challenge to the visibility and impact of Brazilian contributors in international academic discourse. Addressing this issue could involve promoting a culture of publication in English and encouraging collaborative projects that facilitate the dissemination of Brazilian research to a broader audience.

### Geographic distribution of the most cited Brazilian-based scientists

**Figure 1:**
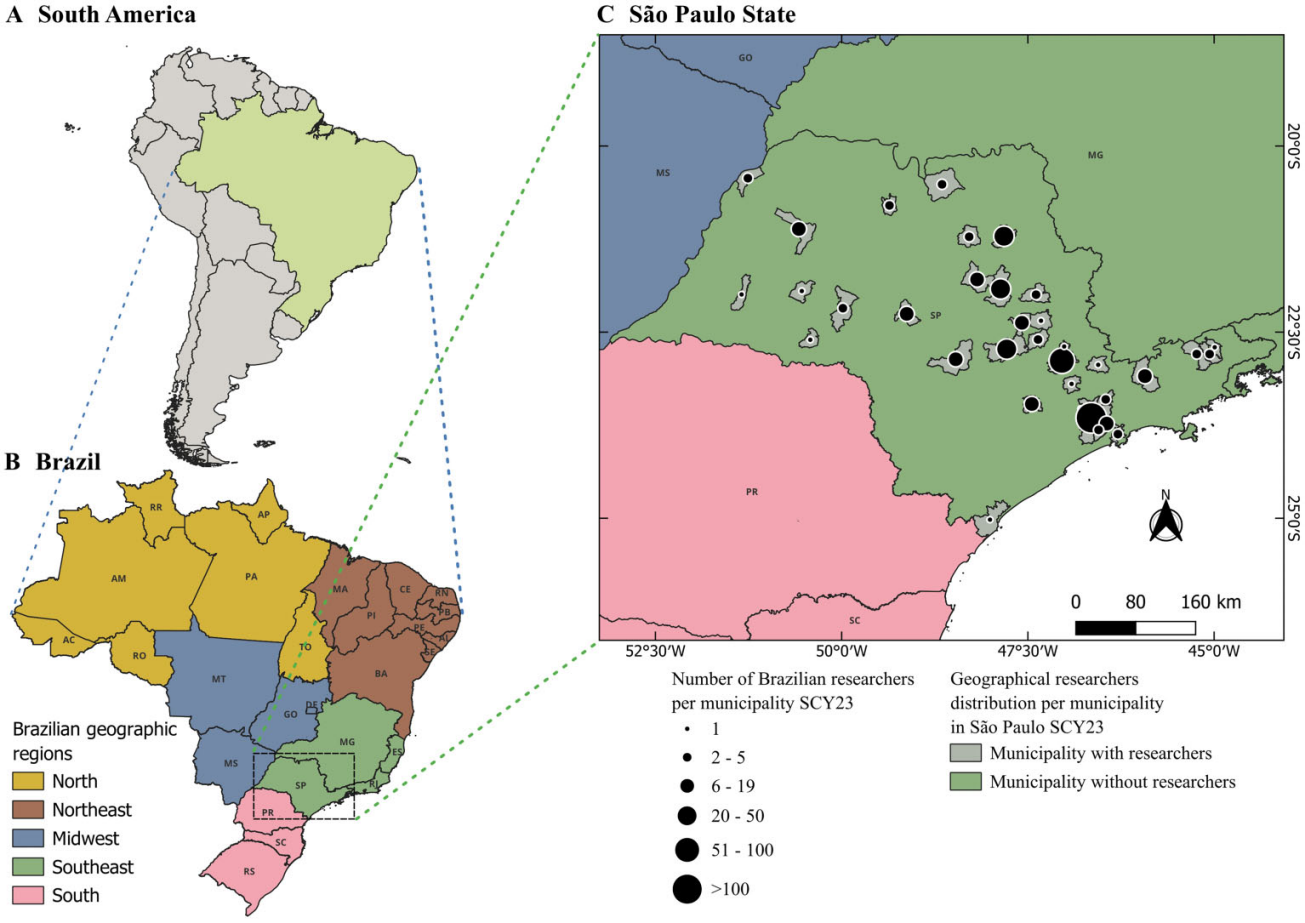
Distribution of highly cited scientists across municipalities of São Paulo State for the single calendar year 2023 (SCY23) database. **A**, A comparison of Brazil's territory to that of South America. **B**, Geographic regions and states of Brazil. **C**, São Paulo State stands out as the region with the highest concentration of highly cited scientists in Brazil, accounting for over 40% of the total. This illustration highlights a prominent technological corridor, underscoring the significant distribution of researchers throughout the state.

The geographic distribution of highly cited scientists within the Brazilian territory surfaces the country's significant disparity. The average proportion of researchers affiliated with institutions in each of the five geographic regions (see map in Figure 1B), together with their percentage to the total in the SCY lists, is as follows: 2.09±0.29% for the North region; 4.36±0.20% for the Midwest; 10.56±0.34% for the Northeast; 17.60±0.17% for the South; and 65.39±0.70% for the Southeast. Analysis of the CL lists shows a similar pattern (Supplementary Tables S16-S25). The consistent stability in both the number and proportion of scientists from each geographic region across the SCY and CL lists indicates a scenario of low mobility among productive scientists in Brazil over time. These findings suggest that efforts to increase the number of graduate programs - responsible for roughly 90% of Brazil's scientific output (21) - and to improve the distribution of research groups nationwide have had minimal impact on reducing the geographic concentration of highly cited researchers. Notably, the Southeast region still hosts over 65% of the most cited Brazilian scientists from the SCY list, with only a slight decrease of less than 5% in the CL lists.

From an economic standpoint, these numbers directly reflect the GDP and long-term public funding for science and technology in each geographic region or state. Remarkably, São Paulo, Rio de Janeiro, and Minas Gerais (all located in the Southeast region) rank as the first, second, and third highest GDPs in the country, respectively (22). Together, their combined GDP accounts for over 50% of the national total, demonstrating a positive correlation with the proportions observed in the CL and SCY evaluations. For example, São Paulo State represents over 30% of Brazil's GDP and comprises an average of 43.82±1.23% of the researchers listed in the CL databases. Likewise, Rio de Janeiro State, which represents approximately 11% of Brazil's GDP, has 16.09±0.95% scientists ranked in the same lists. Minas Gerais State, contributing with around 9% of GDP, has 8.77±0.41% scientists ranked.

São Paulo leadership in these parameters results from decades of substantial investment in science, technology, and innovation, with significant support from São Paulo Research Foundation (FAPESP), a public institution established in 1962 that receives a minimum of 1% of the state's annual tax revenue, as mandated by the State Constitution (23). The stable funding and autonomy of the foundation, together with the designation of 9.57% of the state's annual tax revenue to support the salaries and infrastructure of the three largest state universities, have fostered a strong research network of public universities in São Paulo. This virtuous circle has spurred the growth of nearby cities, creating the so-called ‘technological corridor’, a macroregion characterized by a high or very high HDI (Figure 1C). This also explains why São Paulo State is the only one among the 27 federative units where institutions located in the state capital have less than 50% of the state scientists ranked in the CL list. For example, considering the CL23 list, 191 (41.25%) out of 463 São Paulo-based scientists are affiliated with institutions located in the state capital. Conversely, in Rio de Janeiro and Minas Gerais, most highly cited scientists are affiliated with institutions located in state capitals: 129 (82.17%) out of 157 Rio de Janeiro-based scientists, and 60 (65.93%) out of 91 Minas Gerais-based scientists. Likewise, the research foundations of Rio de Janeiro (FAPERJ) and Minas Gerais (FAPEMIG), founded in 1980 and 1985, respectively, have faced periods of budget scarcity.

**Figure 2:**
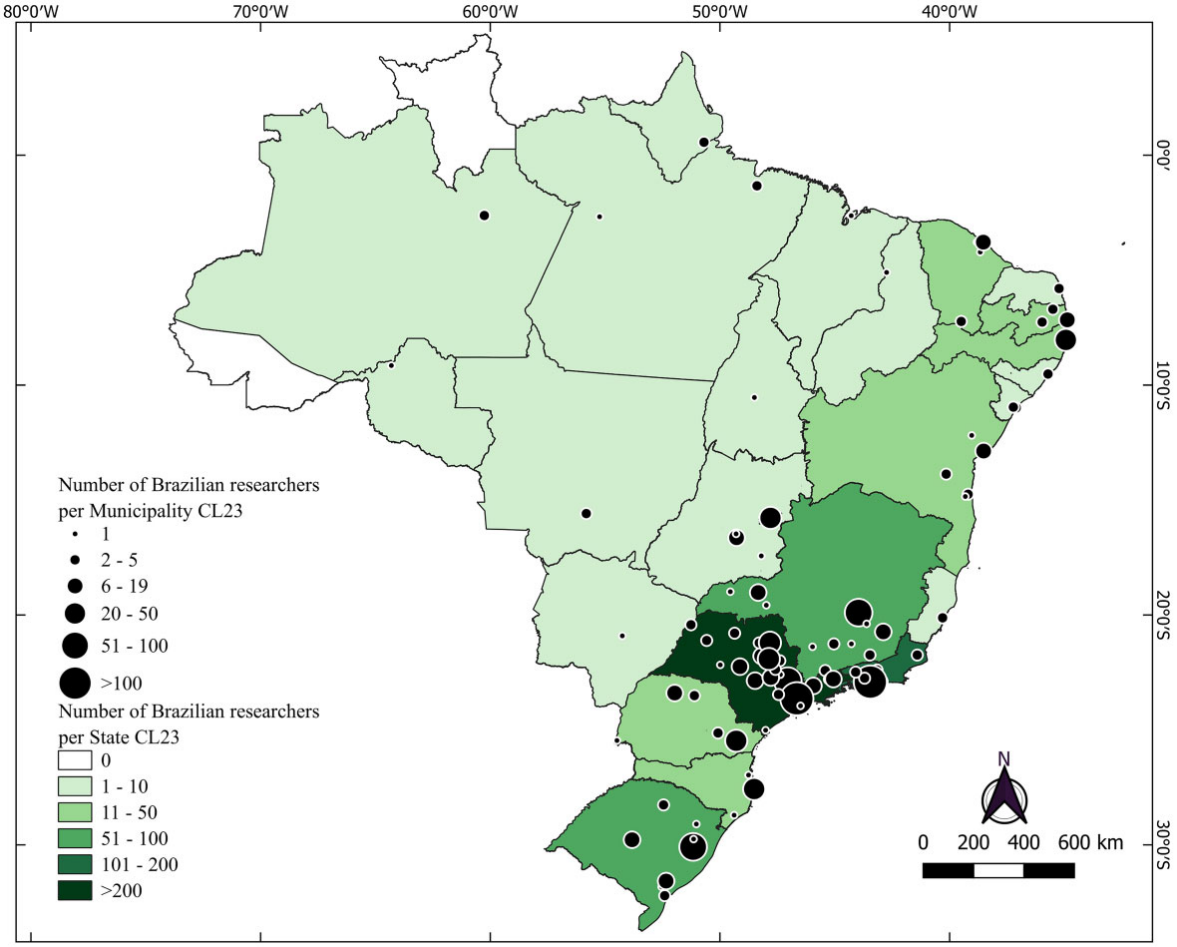
Distribution of highly cited scientists across Brazilian municipalities and states for the career-long 2023 (CL23) database. The figure highlights a significant concentration of researchers in the southeastern and southern regions, in contrast to the lower numbers in the North, Midwest, and Northeast regions.

The clustering of ranked scientists affiliated with institutions located in state capitals is even more pronounced in other Brazilian states. In 13 out of 25 states with scientists ranked in the CL23 list, all listed scientists are from capital-based institutions. Moreover, two out of the 27 states do not have any representatives in the CL23 list (Acre and Roraima), regardless of their institution location (capital or countryside). The CL23 list was used because it had the highest number of states represented (Figure 2), although similar findings were observed in CL lists from previous years. Detailed descriptions of the geographic distribution are provided in Supplementary Tables S26-S45.

### Research productivity grants and types of affiliation

In Brazil, the CNPq supports some of the country's researchers through the PQ award program (17), which aims to recognize and promote the scientific and technological production of investigators. Currently, the program awards around 16,000 grants to researchers and evaluates various dimensions of their work, including scientific output, mentorship, and training of students, and participation in research projects. Recipients receive a fellowship along with a small associated grant designated for research activities. Although some members of the scientific community criticize the criteria used by the CNPq committees and may choose not to apply for the PQ award, the call for proposals is open to all PhD holders involved in scientific activities across the country. To explore the relationship between this support and researcher prominence, the list of PQ grantees (information available in the Lattes CV) was cross-referenced with the Brazilian scientists ranked in the CL lists to assess the extent of overlap between these databases.

**Figure 3:**
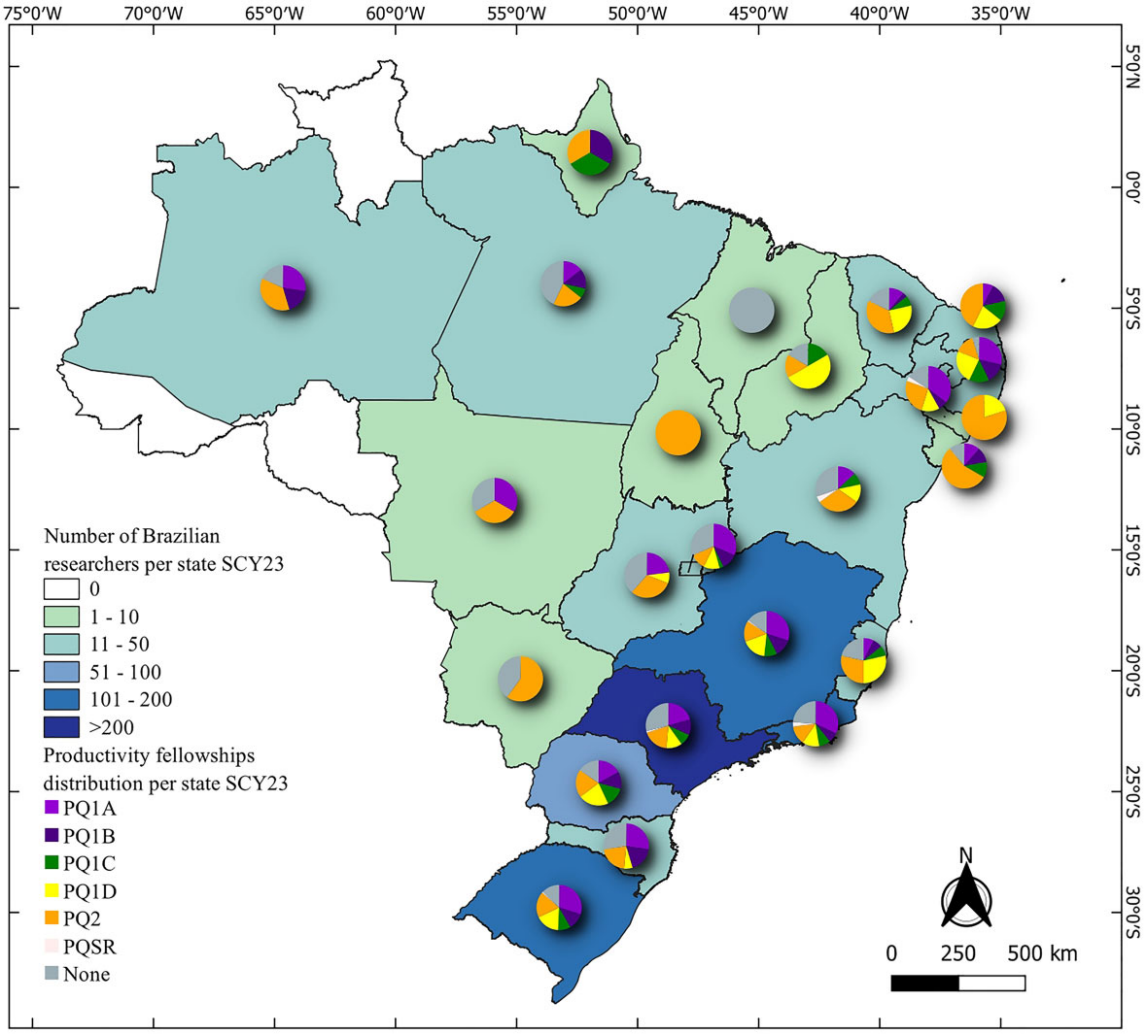
Distribution of research productivity awardees per state for the single calendar year 2023 (SCY23) database. The pie chart illustrates the distribution of research productivity fellowships (PQ) awarded across the states. The SCY23 database was chosen as it represents the most recent data due to the periodical renewal of the PQ awards.

The average percentage of PQ recipients among the most cited researchers in the SCY lists was 73.94±2.38%. Of note, the CL lists were not used for this comparison due to the high number of deceased or retired researchers who are no longer PQ recipients. We estimate that approximately 25-30% of the discrepancies between the databases are due to other parameters considered by evaluation committees when classifying research productivity in Brazil, such as the number of graduate students supervised, involvement in scientific societies and associations, and organization of scientific meetings (Figure 3).

When examining the type of affiliation associated with the researchers ranked in the CL and SCY databases, we found a clear predominance of public institutions over private ones. The average number of affiliations with public institutes, and the corresponding percentage relative to the total number of researchers, was 92.57±0.37% for Brazilian-based scientists ranked in the CL lists. This pattern was also evident in the SCY lists, with 90.50±0.68% researchers affiliated with public institutions. The approximate 2% difference between the CL and SCY lists was too subtle to infer a significant shift of productive scientists toward private institutions, although longitudinal studies over the next decade will provide a clearer picture of this trend. Except for a handful of private institutions, most productive researchers in Brazil are affiliated with public organizations. According to the Brazilian Ministry of Education's 2024 Higher Education Census, out of 2,561 higher education institutions nationwide (the major source of scientific production), 2,244 are private, while 317 are public (24). These data raise concerns about the rapid expansion of private institutions in Brazil, which experienced significant growth since the mid-1990's. However, this expansion does not appear to have resulted in a corresponding increase in the qualified production of knowledge. This can be attributed to a combination of factors predominantly present in the environment of Brazilian public institutions, such as a high proportion of PhD holders among faculty, elevated levels of academic and scientific prowess, intense job competition, the use of research achievements for promotion and career advancement, a significant number of graduate programs, substantial government funding and investment in infrastructure, and international collaborative ventures and initiatives.

### Brazilian performance compared with other South American countries

The number of Brazilian scientists included in the lists surpasses the total number of scientists from all other South American countries combined, regardless of the year and citation assessment considered (CL or SCY). Specifically, for CL19, CL20, CL21, CL22, and CL23, the number of South America-based scientists was, respectively, 965, 1,268, 1,333, 1,503, and 1,654. Brazil-based scientists consistently represented an average of 62.05±1.05% of these totals over the years. A similar pattern was observed in the SCY lists, which had 1,256 (SCY19), 1,676 (SCY20), 1,793 (SCY21), 1,912 (SCY22), and 1,979 (SCY23) South America-based scientists, with Brazil-based scientists making up about 66.50±0.17% of this group. It is worth noting that countries such as Guyana and Suriname had no representatives in either the CL or SCY lists. Furthermore, six countries - Venezuela, Uruguay, Peru, Paraguay, Bolivia, and Ecuador - accounted for less than 6% of the ranked researchers in South America. The remaining countries - Brazil, Argentina, Chile, and Colombia - together comprise approximately 94% of the most cited researchers affiliated with South American institutions.

Brazil also stands out among South American countries in other parameters. In addition to having nearly 50% of the population and 47% of the territory (see map in Figure 1A), Brazilian expenditure on Research and Development (R&D) as a percentage of GDP (RD%GDP) is the highest among its neighbors at 1.15%. The next highest RD%GDPs are from Argentina, Uruguay, and Chile, at 0.54, 0.45, and 0.33%, respectively (Supplementary Table S46). Despite these encouraging numbers, Brazil lags behind other South American countries in terms of the number of researchers relative to population size. While Brazil has 4.9 researchers among the most cited for every 1 million inhabitants (4.9:1M ratio) in CL23, Chile has 10.9:1M, Uruguay 5.9:1M, and Argentina 5.3:1M.

### Brazilian performance compared to the rest of the world

Brazil falls behind countries with higher GDP per capita and HDI from North America, Europe, Asia, and Oceania, regardless of population or economy size. For instance, across all years and types of lists, the United States and Canada have a significantly larger number of highly cited scientists than Brazil. Some European countries, including the United Kingdom, Germany, France, Italy, Sweden, and Switzerland, also boast a much larger number of scientists in their databases relative to their population sizes.

**Figure 4:**
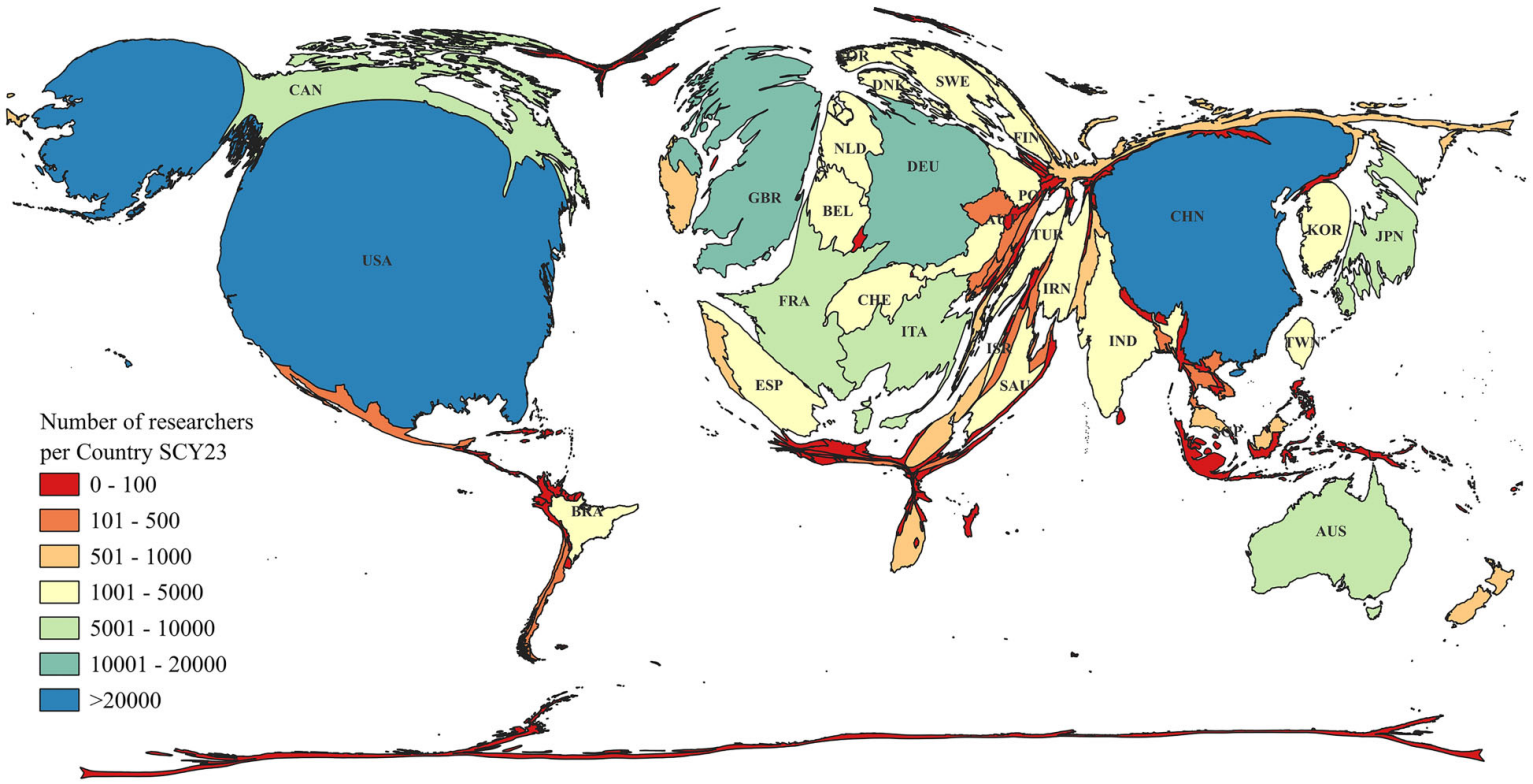
Anamorphic map illustrating the distribution of highly cited scientists in the single calendar year 2023 (SCY23) database. The map distorts the geographical areas of countries proportionally according to the number of researchers in each location. This visualization emphasizes the substantial concentration of researchers in the northern hemisphere; for example, the African continent appears significantly smaller than its actual size, while Latin America is predominantly represented by Brazil. Oceania maintains its proportional size, while Europe appears larger.

Similarly, several Asian countries with high HDI, such as Japan, South Korea, and Israel, also have a greater number of scientists listed under the CL and SCY parameters. In Oceania, Australia has many more ranked scientists than Brazil, while New Zealand has comparable numbers, despite having a population less than 3% of Brazil's. Notably, all the aforementioned countries from these four continents have smaller populations than Brazil, except for the United States. In Africa, except for South Africa, no other country features a significant number of scientists in the CL or SCY lists when evaluated individually (Figure 4).

### Final remarks

Brazil is currently the 7th most populous country, has the 5th largest territory, and typically ranks among the top ten largest economies in the world (25). However, significant inequalities in development persist, as Brazil ranks 87th in HDI, 80th in GDP per capita, 61st in extreme poverty rates, and between 50th and 60th in the Program for International Student Assessment (PISA). These disparities are also evident in Brazilian science. Data and comparisons highlight that while Brazilian science exhibits notable strengths relative to its continental neighbors, it falls short compared to more developed nations. Geographical discrepancies within the country are particularly pronounced, with the three wealthiest states accounting for nearly 70% of the most cited Brazilian-based scientists. Given the complexity of this topic, we offer this critical evaluation to administrators and legislators to provoke further discussion: I) What are the prospects for Brazilian science? II) In which strategic areas should the country focus its investments? III) How can the historical concentration of scientific production in Brazil be effectively addressed?

Brazilian scientists have long advocated for new national science policies and targeted investments that consider the country's natural vocation and specific needs. In closing, paraphrasing the Red Queen's words to Alice in Lewis Carroll's classic Through the Looking Glass, if Brazil truly aspires to become a scientific power, its current pace is far from ideal: “*Now, here, you see, it takes all the running you can do, to keep in the same place. If you want to get somewhere else, you must run at least twice as fast as that!*” (26). By fostering stronger engagement with the global research community, Brazilian scholars can enhance their visibility and recognition across diverse fields, thereby enriching the national and international academic landscapes. To advance in this direction, it is essential to prioritize long-term stable investments that address the country's unique needs and harness its full potential for innovation - particularly in underexplored strategic fields and subfields. Strengthening collaboration within these areas will not only help align research efforts with Brazil's natural vocations and societal demands but also position the country as a leader in scientific excellence and innovation on the global arena.

### Study limitations

This study is primarily based on citation metrics, which may not fully capture the quality or societal impact of research in Brazil. In this regard, an important consideration influencing the distribution of researchers in the CL and SCY databases is the well-documented imbalance in scientometric parameters of papers published by researchers affiliated with institutions in Brazil and, more broadly, across Latin America and other regions of the Global South. This multifaceted issue is shaped by structural, economic, and cultural factors, including funding disparities, research infrastructure, and the already-mentioned language barriers (27). A previous study compared the impact factor performance of articles from Latin American countries (Brazil, Argentina, Chile, and Mexico) with those from five developed nations (USA, England, France, Germany, and Japan) in a set of scientific journals indexed by Web of Science. Strikingly, the findings revealed that while collaborative articles, particularly those involving authors from developed countries, achieved higher visibility and citation rates, non-collaborative articles from Latin America received significantly fewer citations, despite being published in reputable journals (28). Addressing this asymmetry requires efforts to enhance research funding, strengthen institutional capacity, and foster collaborative networks; however, the essential step is to dismantle the biases that hinder the visibility of research from Brazil and the Global South (29,30). Several initiatives, such as the Declaration on Research Assessment (DORA) (31), the Leiden Manifesto (30), the Coalition for Advancing Research Assessment (CoARA) (32), and the Helsinki Initiative on Multilingualism in Scholarly Communication (33), have emerged to address quality assessment in science. These initiatives encourage equitable, holistic, and inclusive publishing practices and propose reforms aimed at improving research outputs and academic communication. Additionally, despite manual verification of Brazilian scientists' affiliations and careers using Lattes CVs, some errors or nuances, such as dual or transient affiliations, may persist. The analysis focuses on highly cited researchers, representing only a small segment of Brazil's scientific community, and may not reflect contributions from early-career or mid-tier scientists. Moreover, disparities across regions and fields/subfields are shaped by historical investment patterns and socioeconomic factors, limiting causal interpretation and the effectiveness of specific policy measures.

## Data Availability

All data generated or analyzed during this study are included in this published article or are available from the corresponding author on request.
