# Supplementary material for: Brazilian science through the looking glass: a scientometric perspective from within and beyond
Source: Braz J Med Biol Res. 2026 Apr 27;59:e15002. doi: 10.1590/1414-431X2026e15002 (PMC13124034; doi:10.1590/1414-431X2026e15002)
Supplement: Supplementary Material [file 1414-431X-bjmbr-59-e15002-suppl.pdf]

Table S46. Research metrics comparison by country.

Country	CL 19		CL 20		CL 21		CL 22		CL 23		RD%GDP[1]	HDI[2]	GDP/cap[3]
	Res	Res/1M	Res	Res/1M	Res	Res/1M	Res	Res/1M	Res	Res/1M			
Brazil	582	2.7	791	3.7	819	3.8	945	4.4	1040	4.9	1.15%	0.760	10295
South America													
Argentina	171	3.8	204	4.5	223	4.8	226	4.8	240	5.3	0.54%	0.849	14187
Chile	127	6.6	156	8.0	164	8.4	192	9.7	214	10.9	0.33%	0.860	17068
Colombia	33	0.6	42	0.8	44	0.8	54	1.0	55	1.0	0.29%	0.758	6947
Venezuela	18	0.6	24	0.8	25	0.8	25	0.8	23	0.8		0.699	0
Peru	10	0.3	17	0.5	24	0.7	19	0.5	25	0.7	0.17%	0.762	7907
Uruguay	13	3.7	15	4.3	19	5.5	20	5.8	20	5.9	0.45%	0.830	22798
Ecuador	7	0.4	13	0.7	13	0.7	16	0.8	21	1.1		0.765	6610
Paraguay	0	0	2	0.3	2	0.3	2	0.2	2	0.3	0.16%	0.731	6276
Bolivia	0	0	0	0	0	0	0	0	1	0.08		0.698	3686
Guyana	0	0	0	0	0	0	0	0	0	0		0.742	20765
Suriname	0	0	0	0	0	0	0	0	0	0		0.690	5494
World													
Switzerland	2546	296.9	2974	344.2	3075	353.3	3261	371.8	3431	386.0		0.967	99565
Sweden	2546	247.6	2132	205.9	3033	291.1	3200	305.1	3353	318.2	3.49%	0.952	55517
United Kingdom	15001	224.4	17240	256.9	18166	271.0	18694	279.1	19648	287.5	2.93%	0.940	49464
Australia	5441	214.7	6369	248.2	6562	255.4	7003	269.5	7448	279.4		0.946	64821
United States	68020	207.1	75900	228.9	78014	234.9	81155	243.5	84202	251.4	3.47%	0.927	82769
Canada	7225	192.1	8129	213.8	8360	218.6	8815	226.4	9265	231.1	1.89%	0.935	53431
Israel	1630	180.0	1869	202.8	1932	206.3	2037	213.2	2093	214.5	5.71%	0.915	52642
New Zealand	803	161.2	974	191.3	1000	195.6	1076	209.9	1132	216.7		0.939	48281
Germany	8792	105.8	9987	120.0	10746	129.1	10989	130.7	11572	138.9	3.13%	0.950	54343
France	5048	74.9	5916	87.5	6393	94.3	6522	96.0	6921	101.3	2.28%	0.910	44691
Italy	4008	67.1	4956	83.3	5397	91.3	5722	97.2	6271	106.3	1.51%	0.906	39003
Japan	6316	49.8	7362	58.3	7557	60.1	8126	64.9	8261	66.3	3.27%	0.920	33767
South Korea	1350	26.0	1750	33.7	1881	36.3	2120	41.0	2366	45.7	4.8%	0.929	33121
South Africa	536	9.2	700	11.9	752	12.6	775	12.9	827	13.1	0.6%	0.717	6023
China	5272	3.7	7795	5.5	16604	11.7	9013	6.3	10687	7.6	2.41%	0.788	12614
Russia	709	4.9	910	6.3	959	6.6	906	6.3	980	6.8	1.09%	0.821	13817
India	1491	1.0	2273	1.6	3352	2.3	2578	1.8	2939	2.0	0.65%	0.644	2481

[1]Research and development (RD) as a percentage of gross domestic products (GDP) in 2020; [2]Human Development Index (HDI) in 2022; [3]GDP per capita (current US$) in 2023. CL: career-long citation impact; Res: Researchers; Res/1M: Number of researchers per 1 million inhabitants.
